# Supplementary material for: Quality assuring the quality assurance tool: applying safety-critical concepts to test framework development
Source: PeerJ Comput Sci. 2022 Oct 28;8:e1131. doi: 10.7717/peerj-cs.1131 (PMC9680868; doi:10.7717/peerj-cs.1131)
Supplement: Supplemental Information 1 — Here we suggest a possible application of the results as an augmented agile process with added rigour through mini V-models controlled by case-specific Definitions of Done. [file peerj-cs-08-1131-s001.pdf]

Appendix A - supplementing:
Quality assuring the quality assurance tool: Applying safety-critical concepts to test framework development

Jonathan Thörn[1], Per Erik Strandberg[1,2], Daniel Sundmark[2], and Wasif Afzal[2]

[1]**Westermo Network Technologies AB, Västerås, Sweden**
[2]**Mälardalen University, Västerås, Sweden**

A CASE-SPECIFIC DEFINITION OF DONE PROPOSAL

This appendix contains the proposed case-specific Definition of Done (DoD) for the three fundamental phases of planning[1], development and testing. Each DoD lists activities to be completed before transition to the following phase. Each DoD only acts as a gate in the development, but does not prescribe when activities are to be performed. Thus, flexibility in development is to some extent maintained.

The DoD for each phase acts as a gate, listing activities to be completed before a task may transition to the next phase. Based on both the related work and the presented candidates, we propose an agile process augmented with influences from safety-critical development. The defined phases and activities in the Definition of Done can be seen as a process controlling document, rather than as guidelines only. With the verification link between the planning phase and the test phase, we argue for a sequence of *mini-v:s* – a V-augmented agile development in three phases as illustrated in Figure 1.

A.1 Suggested Supporting Documentation

The focus group identified a need for supporting documentation. Thus, identified activities listed in the DoDs may refer to one or more of the following supporting documents: (1) Guidelines for branching in source code version-control systems, (2) Guidelines for writing and documenting requirements, (3) Development guidelines, (4) Coding style, (5) Development checklist, (6) Guidelines for documentation, as well as (7) Guidelines for conducting peer-reviews.

A.2 Planning Phase Definition of Done

The suggested activities to be completed during the planning phase, before transitioning to the development phase, are:

P1 Branch(es) created. Branching is done at an early stage to enable documentation of requirements during planning, and to isolate it from the stable framework branch.

P2 Proposed change(s) clarified as "top-level" requirements. The proposed changes may come from the Westermo Operating System (WeOS) teams during their planning phase, identified as new or altered functionalities in the framework, or come from within the test team. Here a requirement means a statement describing a functionality that is expected by the system based on the proposed change.

P3 Important framework interaction sequences identified. The purpose of this activity is to describe expected functionality at lower levels, enabling requirement decomposition and allocation, and to ease identification of possible errors and abnormal operating conditions in later risk analysis. The interactions should be based on the expected functionality.

P4 Third party functionalities identified and suitable libraries and tools selected. To avoid the use of too many tools (e.g. packet generators), using a tool already in successful use, could be tried before adding a new tool. If a new tool/library is needed, its history should be reviewed and the basis for selection documented.

[1]The DoD of the planning phase could be seen as largely overlapping with the agile construct of Definition of Ready, i.e. they define how well requirements have to be specified before development can begin.

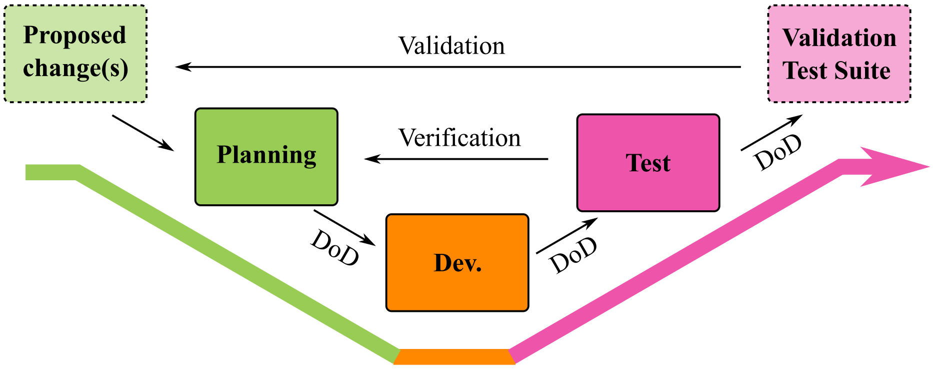

Figure 1. A suggested mini V-model controlled by DoDs.

P5 Preliminary risk and impact analysis performed and documented. Utilize the interaction sequences of P3 to identify possible errors and their effects, including internal errors in the framework as well as errors caused by abnormal operating conditions.

P6 Lower level requirements elicited and allocated to framework components. Break down requirements into smaller workable and testable units. Allocate these to framework components (e.g. modules/tools/tool-chains), according to identified interaction sequences. Also, requirements derived from the risk analysis should be allocated to suitable components.

P7 Development impediments identified and mitigated. Identify factors that may block or delay the development, and find mitigation strategies.

A.3 Development Phase Definition of Done

The suggested activities to be completed during the development phase, before transitioning to the test phase are defined in the provided list below.

D1 Complete implementation according to requirements and development guidelines. Avoidance of faults being introduced by misconceptions, defining e.g. conventions, error handling, and other practises. These could be combined with development checklists to reduce the effort for later reviews.

D2 Static code analysis only giving "low level" remarks. Linting[2] and/or other static analysis tools, e.g. Coverity[3], should be set up to the development branch to enable continuous correction during development.

D3 Unit tests written. To test fine-grain logic, unit tests of developed components should be written and refined before, during and after the implementation is performed.

D4 Tests written to verify compliance with requirements. In parallel to the implementation, tests to verify compliance with requirements should be developed.

D5 Behaviour, instructions and constraints defined in documentation. Proper documentation of system behaviour, usage instructions and system constraints to be ensured.

D6 Peer-reviews completed and documented. Definition of methods for peer review, which should include examination of the implementation, tests and documentation.

D7 Issues found by peer-reviews corrected. After correction, this should be verified with the reviewer.

A.4 Test Phase Definition of Done

The suggested activities to be completed during the test phase, before transitioning to the tool validation test-suite and subsequent merge of the new functionality with the maintained stable solution, are defined in the provided list below.

T1 Unit tests performed. Verify low level behavior by running the newly developed unit tests (this may be an iterative process, see DoD D3).

T2 Unit-integration tested. Test the integration of units as a group, as well as the data transfer between components.

[2]Linting is brought up in Section 5.2.2.
[3]`https://scan.coverity.com`

T3 Requirement-based tests performed. These tests verify the expected functionality of the framework as described by the requirements.

T4 Fault injection tests performed. Fault-injection can be used in two ways, for two purposes. (i) Forced errors in components/sub-tools of the tool-chain can verify the error detection or prevention measures in other parts of the system (e.g. monitoring services, sanity checks, etc.). (ii) Faults could also be introduced in the WeOS code, and when running test cases for WeOS, we expect the framework to detect the problems (test cases should fail).

T5 All detected issues managed. After correction, applicable tests should be repeated for verification of sufficient correction.

T6 Risk and impact analysis documentation completed. The documentation should be revisited and completed. If necessary, a new analysis can be conducted to validate sufficiency of implemented measures.
