# Supplementary material for: Quality assuring the quality assurance tool: applying safety-critical concepts to test framework development
Source: PeerJ Comput Sci. 2022 Oct 28;8:e1131. doi: 10.7717/peerj-cs.1131 (PMC9680868; doi:10.7717/peerj-cs.1131)
Supplement: Supplemental Information 2 — This document answers the checklist described in Tong, A., Sainsbury, P., & Craig, J. (2007). Consolidated criteria for reporting qualitative research (COREQ): a 32-item checklist for interviews and focus groups. International journal for quality in health care, 19(6), 349-357. The checklist is the substitute for the raw data which cannot be published. [file peerj-cs-08-1131-s002.pdf]

Consolidated criteria for reporting qualitative research (COREQ)

This document answers the checklist described in Tong, A., Sainsbury, P., & Craig, J. (2007). Consolidated criteria for reporting qualitative research (COREQ): a 32-item checklist for interviews and focus groups. *International journal for quality in health care*, *19*(6), 349-357. The answers cover the submission of *Quality assuring the quality assurance tool: A case study on applying safety-critical concepts to test framework development* to PeerJ in early 2022, by Jonathan Thörn[1], Per Erik Strandberg[1,2], Daniel Sundmark[2], and Wasif Afzal[2]. (1: Westermo Network Technologies AB, Västerås, Sweden. 2: Mälardalen University, Västerås, Sweden).

Domain 1: Research team and reflexivity

Personal Characteristics

1. Interviewer/facilitator

Which author/s conducted the interview or focus group?
The first author was responsible for preparing and running the presentation, and further to introduce and explain presented activities, concepts, and candidates. The second author acted as moderator during discussions, i.e. by keeping track of coverage regarding both topics and speakers, and asking for further elaborations when necessary while trying to maintain fluent and self-driven conversations. The third author acted as support of the execution by clarifying the purpose, assisting in understanding the purpose, and would step in if needed to keep the activities in line with the goals.

2. Credentials

What were the researcher's credentials? E.g. PhD, MD
First author: M.Sc.Eng student
Second author: Licentiate.
Third and fourth author: Professor

3. Occupation

What was their occupation at the time of the study?
First author: M.Sc.Eng student, during master's thesis.
Second author: Industrial doctoral student employed at the industry partner.
Third and fourth author: Professor.

4. Gender

Was the researcher male or female?
All authors are male.

5. Experience and training

What experience or training did the researcher have?
First author: Several courses on research, and research methods.
Second author: Had conducted several empirical studies.
Third and fourth author: Leaders of a research group on software testing, and research methods educators.

Relationship with participants

6. Relationship established

Was a relationship established prior to study commencement?
Yes. The second author had been employed at the company for several years, and had conducted several prior research studies with the third and fourth authors involving the company. The first author had worked as a summer intern at the company, and was working on his master's thesis on site.

7. Participant knowledge of the interviewer

What did the participants know about the researcher? e.g. personal goals, reasons for doing the research
The participants were aware that the research was conducted as part of the first author's master's thesis. Further, the participants knew that the research involved an overview of internal processes.

8. Interviewer characteristics

What characteristics were reported about the interviewer/facilitator? e.g. Bias, assumptions, reasons and interests in the research topic
The background of the researchers was explained to the participants during the introductory phase of the focus group session. Also, e.g. that the field of the master's thesis was dependable systems. However no specific biases were discussed.

Domain 2: study design

Theoretical framework

9. Methodological orientation and Theory

What methodological orientation was stated to underpin the study? e.g. grounded theory, discourse analysis, ethnography, phenomenology, content analysis
Data collected from the focus group meeting was twofold. First, a quantitative score of how useful the candidates were perceived (categorized as a good idea with high value, indifferent/ambiguous opinion, or unappreciated idea with little or no value.), and the discussions were analyzed with methods similar to thematic analysis. However, this thematic analysis was not explicitly stated in the paper.

Participant selection

10. Sampling

How were participants selected? e.g. purposive, convenience, consecutive, snowball
Aiming at diversity in terms of experience and specialization, we recruited a stratified convenience sample of six individuals for the focus group: one manager for the software test team who is responsible for the framework, one manager for a software development team, three developers from the test team, as well as one developer from the software development team.

11. Method of approach

How were participants approached? e.g. face-to-face, telephone, mail, email
The second author invited participants after face-to-face discussions.

12. Sample size

How many participants were in the study?
Six.

13. Non-participation

How many people refused to participate or dropped out? Reasons?
One of the invited participants did not participate. Also, the software developer had to leave before the quantitative part took place, which was at the very end of the session.

Setting

14. Setting of data collection

Where was the data collected? e.g. home, clinic, workplace
The focus group was conducted in a conference room at the company site. Two participants join remotely over a video link.

15. Presence of non-participants

Was anyone else present besides the participants and researchers?
No.

16. Description of sample

What are the important characteristics of the sample? e.g. demographic data, date
The focus group was conducted as one single session in early April 2020. Further, one participant was female, the remaining were male. See also question 10.

Data collection

17. Interview guide

Were questions, prompts, guides provided by the authors? Was it pilot tested?
The focus group was guided by a presentation, which was created by the first author and further reviewed and refined by the second and third authors. Also, a discussion guide was prepared before the focus group execution, which contained additional and more elaborate questions to stimulate the discussions if needed. No pilot was conducted.

18. Repeat interviews

Were repeat interviews carried out? If yes, how many?
No.

19. Audio/visual recording

Did the research use audio or visual recording to collect the data?
No.

20. Field notes

Were field notes made during and/or after the interview or focus group?
All qualitative data collection was conducted by taking notes during the discussions. The field notes were taken on prepared templates. To mitigate risks of missed aspects or misconceptions regarding the content and

implications from discussions, redundancy in notes was provided by having all three execution roles simultaneously taking notes throughout the focus group session.

21. Duration

What was the duration of the interviews or focus group?
The focus group meeting was three hours.

22. Data saturation

Was data saturation discussed?
No.

23. Transcripts returned

Were transcripts returned to participants for comment and/or correction?
No.

Domain 3: analysis and findings

Data analysis

24. Number of data coders

How many data coders coded the data?
The first author analyzed the data.

25. Description of the coding tree

Did authors provide a description of the coding tree?
No.

26. Derivation of themes

Were themes identified in advance or derived from the data?
The main aspects (development, analysis, run-time measures, and validation and verification) as well as the candidates were the scaffold for the data analysis, they had been identified before the focus group. During the data analysis, the three sentiments (good idea, indifferent, or bad idea) emerged.

27. Software

What software, if applicable, was used to manage the data?
Pen and paper, as well as the Google suite (docs, sheets, etc.)

28. Participant checking

Did participants provide feedback on the findings?
No.

Reporting

29. Quotations presented

Were participant quotations presented to illustrate the themes / findings? Was each quotation identified? e.g. participant number
The paper contains some anonymized quotations. In the field notes, each opinion was identified to enable differentiating group opinions from individual opinions by attempting to identify consensus reached within the group.

30. Data and findings consistent

Was there consistency between the data presented and the findings?
Yes. The candidates were identified in section 5, using a literature study. In section 6, we describe how the candidates are validated with a focus group. Table 1 summarizes the findings and provides traces back to previous steps of the analysis.

31. Clarity of major themes

Were major themes clearly presented in the findings?
Yes, in particular in Table 1.

32. Clarity of minor themes

Is there a description of diverse cases or discussion of minor themes?
Partly.
